# Treatment with Class A CpG Oligodeoxynucleotides in Cats with Naturally Occurring Feline Parvovirus Infection: A Prospective Study

**DOI:** 10.3390/v12060640

**Published:** 2020-06-12

**Authors:** Filippo Ferri, Federico Porporato, Francesco Rossi, Daniela Enache, Carolina Callegari, Gabriele Gerardi, Luigi M. Coppola, Barbara Contiero, Chiara Crinò, Neda Ranjbar Kohan, Marina L. Meli, Hans Lutz, Regina Hofmann-Lehmann, Eric Zini

**Affiliations:** 1 AniCura Istituto Veterinario di Novara, Strada Provinciale 9, 28060 Granozzo con Monticello (NO), Italy; federico.porporato@anicura.it (F.P.); francesco.rossi@anicura.it (F.R.); daniela.enache@anicura.it (D.E.); carolina.callegari@anicura.it (C.C.); ezini@vetclinics.uzh.ch (E.Z.); 2 Department of Animal Medicine, Production and Health, University of Padova, viale dell’Università 16, 35020 Legnaro (PD), Italy; gabriele.gerardi@unipd.it (G.G.); luigi.coppola@unipd.it (L.M.C.); barbara.contiero@unipd.it (B.C.); 3 The Ralph Veterinary Referral Centre, Fourth Avenue, Globe Business Park, Marlow SL7 1YG, UK; chiara.crino@theralph.vet; 4 Clinical Laboratory, Department of Clinical Diagnostics and Services, and Center for Clinical studies, Vetsuisse Faculty, University of Zurich, Winterthurerstrasse 260, 8057 Zurich, Switzerland; neda.jamei@gmail.com (N.R.K.); mmeli@vetlabor.ch (M.L.M.); hanslutz@me.com (H.L.); rhofmann@vetclinics.uzh.ch (R.H.-L.); 5 Clinic for Small Animal Internal Medicine, Vetsuisse Faculty, University of Zurich, Winterthurerstrasse 260, 8057 Zurich, Switzerland

**Keywords:** cat, feline parvovirus, CpG-A, treatment, antiviral, Mx protein

## Abstract

Feline parvovirus (FPV) causes severe gastroenteritis and leukopenia in cats; the outcome is poor. Information regarding specific treatments is lacking. Class A CpG oligodeoxynucleotides (CpG-A) are short single-stranded DNAs, stimulating type I interferon production. In cats, CpG-A induced an antiviral response in vivo and inhibited FPV replication in vitro. The aim was to prospectively investigate the effects of CpG-A on survival, clinical score, hematological findings, antiviral response (cytokines), viremia, and fecal shedding (real-time qPCR) in cats naturally infected with FPV. Forty-two FPV-infected cats were randomized to receive 100 µg/kg of CpG-A (*n* = 22) or placebo (*n* = 20) subcutaneously, on admission and after 48 h. Blood and fecal samples were collected on admission, after 1, 3, and 7 days. All 22 cats showed short duration pain during CpG-A injections. The survival rate, clinical score, leukocyte and erythrocyte counts, viremia, and fecal shedding at any time-point did not differ between cats treated with CpG-A (50%) and placebo (40%). Antiviral myxovirus resistance (*Mx*) gene transcription increased in both groups from day 1 to 3 (*p* = 0.005). Antibodies against FPV on admission were associated with survival in cats (*p* = 0.002). In conclusion, CpG-A treatment did not improve the outcome in cats with FPV infection. FPV infection produced an antiviral response.

## 1. Introduction

Feline parvovirus (FPV) is a small, non-enveloped, serologically homogeneous parvovirus with a single-stranded DNA genome. Cats affected by feline panleukopenia present severe hemorrhagic gastroenteritis and leukopenia [1]. The infection is highly contagious and associated with high morbidity and mortality rates, with the latter ranging from 25 to 90% and up to 100% in cats with acute and hyper-acute infections, respectively. The prevalence of subclinical infections remains unknown [2]. Body weight, the severity of clinical signs, thrombocytopenia, hypoalbuminemia, hypokalemia, glucose administration, total T4 have been identified as negative prognostic factors in cats infected by FPV, whereas leukopenia has been inconstantly recognized as a negative outcome predictor [3,4,5].

So far, no specific antiviral therapy against FPV infection has been tested in cats, and its management relies upon supportive treatment. Plasma or whole blood transfusion may be required in case of severe hypoproteinemia or anemia, respectively [3,4,5,6]. Recently, feline interferon regulatory factor-1 has been found to reduce the replication of FPV on Crandell-Rees feline kidney cells [7], but no information is yet available in vivo.

The CpGs oligodeoxynucleotides are bacterial DNA nucleotide sequences containing CpG (cytosine–guanosine) dinucleotide motifs that are under-represented in vertebrate genomes and, usually, present in the methylated form. Due to the different chemical structure, immune cells are able to recognize bacterial CpG, triggering the immune response [8,9]. Indeed, oligonucleotides containing unmethylated CpG (CpG ODN) have been shown to activate the canine, bovine, ovine, murine, and primates’ innate immune system [10,11,12,13,14,15]. The CpG ODNs have been allocated to different classes based on their specific CpG sequence, backbone structure, and immune-modulatory effects; among them, classes A and B are the most studied [16]. In particular, class A CpG ODNs (CpG-A) induces massive type 1 interferon (IFN) secretion by plasmacytoid dendritic cells and increases natural killer’s cytotoxicity, enhancing the innate immunity against viral infections with immunostimulatory properties in mice, primates, and domestic species [16]. Recently, Robert-Tissot and colleagues demonstrated the ability of CpG ODN 2216, a synthetic CpG-A, to induce the transcription of the antiviral myxovirus resistance (*Mx*) gene in cell culture of cats; Mx proteins were GTPases, directly and solely induced by type 1 and 3 IFNs, and their transcription was considered as an indicator of antiviral response in cats [17]. CpG ODN 2216 is able to enhance resistance to feline viruses of five distinct families, namely, Coronaviridae, Herpesviridae, Caliciviridae, Parvoviridae, and Retroviridae [17,18]. Moreover, it induces a systemic antiviral state in healthy domestic cats without any reported side effect [10].

The aim of this study was to investigate the effects of CpG ODN 2216 administration on survival, clinical signs, laboratory findings, antiviral response, viremia, and viral shedding in cats naturally infected with FPV.

## 2. Materials and Methods

### 2.1. Cats

The study was approved by the animal welfare committee of the University of Padua (Prot. n. 152450, 6/5/2016). Cats admitted at the authors’ institution and who were diagnosed with feline panleukopenia were eligible for inclusion. The diagnosis was based on: (1) compatible clinical signs (e.g., hemorrhagic gastroenteritis, hyperthermia or hypothermia, severe dehydration) and/or leukopenia (leukocyte count <3500/µL); (2) positive results to the rapid enzyme immunoassay for the detection of canine parvovirus (CPV) antigen in feces (SNAP^®^ parvo test; IDEXX Laboratories, Westbrook, Maine, USA) [19]. To be included in the study, cats had to meet both criteria. In addition, cats had to be unvaccinated, treatment naïve, and with a body weight >500 g; smaller cats were excluded because repeated blood sampling was deemed unfair and potentially dangerous. Fecal flotation and a rapid enzyme immunoassay for the detection of Giardia antigen on feces (SNAP^®^ Giardia test; IDEXX Laboratories) were performed on admission, to rule out other potential causes for the presenting clinical signs. Cats with concurrent intestinal parasites were excluded from the study.

All cats received supportive treatments based on clinical signs and laboratory findings. Cats receiving plasma or whole blood transfusions were excluded from the study to avoid any bias regarding the results of blood parameters, blood viral load of FPV, or antiviral response assessment.

### 2.2. Treatment Protocol and Data Collection

The study was designed as a double-blinded placebo-treatment randomized prospective study. Each cat was allocated to one of the two treatment groups with a software (Minim; https://www-users.york.ac.uk/~mb55/guide/minim.htm) incorporating a partial minimization procedure to adjust the randomization probabilities between groups and balance for covariates collected at baseline. Among covariates, sex, age, body weight, and leukocyte counts were selected. To establish the sample size, the mortality rate was chosen as the guiding variable [4]. In particular, to achieve an ideal reduction of mortality from 70% to 35% in untreated vs. treated cats, with a level of significance of 0.05 and a power of 0.80, the number of cats needed would have been 31.

CpG ODN 2216 (InvivoGen, San Diego, California, USA) was dissolved in endotoxin-free sterile water for injection to reach a concentration of 500 µg/mL. Cats were randomly allocated to receive 100 µg/kg of CpG ODN 2216 or a corresponding volume of sterile saline solution, subcutaneously between shoulders—on admission and after 48 h; both solutions were administered at room temperature.

Breed, age, sex, body weight, and local or systemic adverse reactions associated with the injection (i.e., pruritus, vocalization, pain, erythema, swelling, alopecia, crusts, and systemic signs of a hypersensitivity reaction) were reported on each medical record.

Blood and fecal samples were collected on admission and after 1, 3, and 7 days of hospitalization; complete blood count and blood smear evaluation, quantitative analyses of type 1 IFNs activities, viral load of FPV in the blood (viremia), and semi-quantitative analysis of viral load of FPV in feces (fecal viral shedding) were determined at each time-point. Complete blood count was performed using a commercial analyzer (ProCyte Dx; IDEXX Laboratories), and blood smear evaluation was assessed by a board-certified clinical pathologist. Cats were diagnosed with leukopenia if leukocyte count was <3500/µL and with anemia, if the hematocrit was <27%. In addition, quantification of FPV antibodies was obtained using an immunofluorescence assay, as described on admission in each cat [20]. The antigen preparations used to prepare the slides were tested for the absence of contaminating viruses by RT-PCR and PCR, as previously described [21].

A clinical severity score was developed, combining the cat attitude, appetite, vomiting, and fecal appearance (Table 1).

### 2.3. The Activity of Type 1 Interferons

One-hundred microliters of EDTA-anticoagulated blood were immediately mixed with 300 µL of mRNA lysis buffer (mRNA Isolation Kit I; Roche Diagnostics, Rotkreuz, Switzerland) upon blood collection. The samples were stored at −20 °C within 1 h from the collection. The mRNA was purified using the mRNA Isolation Kit I using the MagNA Pure LC instrument (Roche Diagnostics), according to the manufacturer’s instructions. Two negative extraction controls (1× phosphate-buffered saline (PBS) pH 7.4 (Gibco, ThermoFisher Scientific, Reinach, Switzerland)) were added to each extraction batch (30 samples) to monitor for cross-contamination. The mRNA was eluted into 25 µL of elution buffer and stored at −80 °C until further use. First-strand cDNA was synthesized using the High Capacity cDNA Reverse Transcription Kit (Applied Biosystems, Rotkreutz, Switzerland) according to the manufacturer’s instructions. The TaqMan real-time qPCR assays were performed for the relative quantification of *Mx* gene transcription, as previously described [22]. The calculation of mRNA expression levels from the threshold cycle (Ct)-values and the efficiencies of the cytokine and reference gene assays was performed using GeNorm version 3.5 (qbase+; Biogazelle, Gent, Belgium) [22]. Cytokine transcription levels were normalized to the transcription levels of V-abl Abelson murine leukemia viral oncogene homolog (*ABL*) and zeta polypeptide (*YWHAZ*) genes at each time-point and used to calculate the relative transcription levels compared to day 0 [23]. PCR assays comprised 5 µL cDNA in a total volume of 25 µL per reaction using the TaqMan^®^ Fast Universal PCR Master Mix (Applied Biosystems). All qPCR assays were run concomitantly with a DNA standard using the ABI 7500Fast instrument (Applied Biosystems) with an initial denaturation at 95 °C for 20 s, followed by 45 cycles 95 °C for 3 s and 60 °C for 30 s.

### 2.4. Quantitative Analysis of Viral Loads in Blood and Feces

Two-hundred microliters of EDTA-anticoagulated blood were collected and stored at −20 °C within 1 h of collection. Total nucleic acid (TNA) was extracted from 100 µL blood using the MagNA Pure LC Total Nucleic Acid Kit (Roche Diagnostics), following the instructions of the manufacturer, with an elution volume of 90 µL. Two negative extraction controls (PBS) were concurrently prepared with each batch of samples to monitor for cross-contamination.

For the FPV qPCR assay, a sequence in the high conserved VP-2 gene was amplified using the following primers and probe: PV3294f: 5′ ACTGCATCATTGATGGTTGCA 3′; PV3400r: 5′ GGTATGGTTGGTTTCCATGGA 3′ PV3375p: 5′ FAM CCCAATGTCTCAGATCTCATAGCTGCTGG 6 TAMRA 3′ [24]. Briefly, the 25 µL PCR reaction comprised 12.5 µL of 2× qPCR MasterMix Plus Low ROX (Eurogentec, Seraing, Belgium), 400 nM of each primer, 80 nM of the probe, 0.01 U/µL of UNG (uracil-DNA glycosylase, Eurogentec), and 5 µL of TNA. Assays were performed using an ABI 7500Fast instrument (Applied Biosystems) with an initial UNG activation at 50 °C for 2 min and denaturation at 95 °C for 10 min, followed by 45 cycles 95 °C for 15 s and 60 °C for 1 min.

The single gene feline albumin load was determined using real-time PCR as described [25]. Because FPV-infected cats commonly show severe leukopenia, normalization using housekeeping genes was considered unreliable. Hence, the number of FPV copies was normalized to the volume of blood (mL).

Fecal swabs were collected in sterile tubes and stored at −20 °C within 1 h of collection. Three hundred microliters of PBS were added into the samples; the tubes were vortexed and put on a shaking incubator at 42 °C for 10 min to resuspend the sample. After centrifugation at 8000× *g* for 1 min to remove any liquid from the inside of the lid, the swabs were inverted using a pair of sterilized tweezers and centrifuged again to recover the liquid (freed from the cotton part of the swab) in the bottom of the tube. The swabs were removed, and 200 µL of liquid sample material was used to extract TNA and perform FPV qPCR, as described above for the blood samples. Since it was not possible to quantify the amount of feces on each swab, this analysis was considered semi-quantitative.

Moreover, feline leukemia virus (FeLV) real-time qPCR and FeLV real-time RT-qPCR were performed on TNA purified from EDTA-anticoagulated blood, to investigate the presence of FeLV provirus and viral RNA, as previously described [26].

### 2.5. Antibody and Antigen Detection

Antibody titers to FPV were determined in the serum samples by indirect immunofluorescence (IFA), as previously described [20], in all but four samples that did not have sufficient volume.

FeLV p27 antigenemia and feline immunodeficiency virus (FIV) antibody status of the cats were determined on admission (SNAP FIV/FeLV Combo; IDEXX Laboratories, Westbrook, Maine, USA) to investigate other potential causes that might have influenced the immune response. FeLV results were confirmed using an in-lab double-antibody sandwich ELISA, as described using monoclonal antibodies to three epitopic regions of p27, as previously described [27]. FIV western blot was performed, as described [28]. FeLV and FIV confirmation was possible for all but 4 samples that had insufficient sample volume.

### 2.6. Statistical Analysis

Chi-square test, r × c contingency table, *t*-test, or Mann–Whitney U test was used to compare survival rate, clinical score, leukocyte and erythrocyte count, *Mx* transcription, and FPV DNA loads in blood and in feces in cats treated with CpG ODN 2216 vs. placebo over time. In addition, within each treatment group, comparisons were performed among time-points with repeated measure ANOVA or Friedman test, followed by Dunn’s multiple comparisons test. Datasets were tested for normal distribution using the Shapiro–Wilk test. Thereafter, the entire population of cats was divided into survivors and non-survivors and *Mx* transcription, and FPV DNA loads in blood and in feces were compared between and within the two groups, as described above. Results were reported as median and interquartile range or as percentages. Significance was set at *p* < 0.05. Statistical analysis was performed using the SAS 9.3 commercial software (SAS Institute, Cary, North Carolina, USA).

## 3. Results

### 3.1. Cats, Clinical Data, and Outcome

An outbreak of FPV infection occurred in a large cat shelter located in the north-west of Italy at the end of autumn 2010 and lasted longer than three consecutive years, becoming endemic [3]. Forty-two cats originating from the same shelter had been initially recruited and randomly divided into two groups receiving CpG ODN 2216 (CpG ODN 2216 group) and the placebo (placebo group), respectively. At the time, 22 cats were enrolled in the CpG ODN 2216 group and 20 in the placebo group. Eleven (50%) cats in the CpG ODN 2216 group and eight (40%) cats in the placebo group survived to discharge (*p* = 0.516). Assuming similar outcome rates (i.e., 50% and 40%), in order to achieve a significant difference between CpG ODN 2216 and placebo groups with a power of 0.80, approximately 400 cats would have been needed in each group. Hence, the enrolment of cats was not continued.

All cats were domestic shorthair. Among those in the CpG ODN 2216 group, 9 (40.9%) were female, and 13 (59.1%) were male, with a median age of 3.0 months (interquartile range: 2.0–7.0) and a median body weight of 1.2 kg (interquartile range: 0.8–2.5). Among the cats enrolled in the placebo group, 11 (55%) were female, and 9 (45%) were male, with a median age of 3.5 months (interquartile range: 3.0–6.7) and a median body weight of 1.2 kg (interquartile range: 1.0–1.9). No significant difference was observed regarding sex, age, and body weight between the two groups.

FIV antibody and FeLV antigen tests were performed in 38 cats—19 (81.8%) in the CpG ODN 2216 group, and 19 (95.0%) in the placebo cats. In the four remaining cats, due to the critical clinical conditions on presentation, it was not possible to collect enough blood to perform FIV antibody and FeLV antigen tests. All tested cats were FIV-negative, as confirmed by western blot analysis. All cats were tested for FeLV provirus and viral RNA using molecular assays. Three (15.8%) cats of the treatment group were FeLV provirus-positive, two of them were also viral RNA-positive, and one of them was antigenemic (130% in the ELISA). So, two cats in the treatment group had presumptively regressive FeLV infections, and one cat was undergoing progressive FeLV infection; none of the cats in the placebo group was FeLV-positive in any of the FeLV tests. The scant number of FeLV-positive cats and the absence of FIV-positive cats did not allow any statistical analysis.

No adverse clinical signs were associated with CpG ODN 2216 administration in any cat of the treatment group; however, signs of the pain of short duration (i.e., the vocalization of 1–2 s) were noticed during each injection.

On admission and during hospitalization, depression or lethargy was noticed in 20 (90.9%) cats, treated with CpG ODN 2216, and in 18 (90%) receiving placebo; appetite was decreased in 20 (90.9%) and 19 (95%) cats, respectively. Six (27.3%) cats in the treatment group and nine (45%) in the placebo presented diarrhea on admission, while nine (40.9%) cats in the former and five (25%) in the latter group developed diarrhea during hospitalization. On admission, vomiting was present in four (18.2%) cats in the treatment group and in seven (35%) of the placebo one, while six (27.3%) cats in the former and three (15%) in the latter group developed vomiting during hospitalization.

On admission, the median clinical score of cats treated with CpG ODN 2216 was 6.0 (interquartile range: 5.0–7.5), and of those receiving placebo was 7.5 (interquartile range: 3.3–9.5). The clinical score of cats during hospitalization are reported in Table 2. Although there was a decrease in median values throughout time in both groups, no significant difference was observed within each group. Differences were also not present between the two groups at each time-point.

Sixteen (72.7%) cats in the CpG ODN 2216 group and 14 (70%) in the placebo showed leukopenia on admission, and two cats in each group (9.1% and 10%, respectively) developed leukopenia during hospitalization. Median leukocyte count on admission and during hospitalization are reported in Table 3; no significant differences were found within and between groups at each time-point.

Eleven (50%) cats in the CpG ODN 2216 group and nine (45%) in the placebo were anemic on admission. Two (9.1%) cats in the former group and six (30%) in the latter developed anemia during hospitalization. Plasma or whole blood transfusions were not necessary for any of these cats. Median erythrocyte count on admission and during hospitalization are reported in Table 3; no significant differences were found within and between groups at any time-point.

### 3.2. The Activity of Type 1 Interferons, Blood and Fecal Viral Loads, FPV Antibodies

Cats in both the CpG ODN 2216 and the placebo group had a progressive increase of *Mx* transcription from admission to day 3 of hospitalization, while a decrease was observed from day 3 to 7 (Table 4); the increase of *Mx* transcription was significant at day 3 compared to admission and day 1 in both groups (*p* = 0.005). There were no differences between the two groups at any time-point.

**Figure 1:**
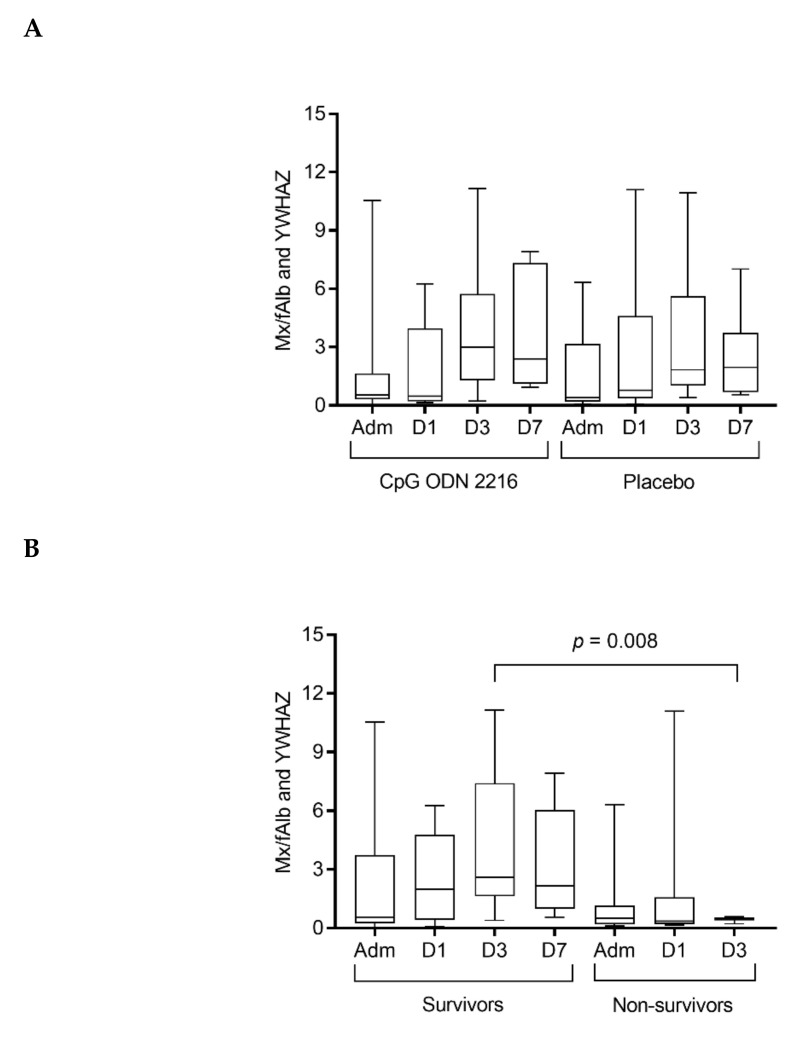
*Mx* (*myxovirus*) transcription in peripheral blood (**A**) in cats treated with CpG ODN 2216 vs. placebo and (**B**) in survivors vs. non-survivors. *Mx* copies were normalized using housekeeping genes—f*Alb* and *YWHAZ*—copies, as determined by real-time qPCR. Box and whisker plot: boxes give median and 25% and 75% quantiles; whiskers extend to min/max. (**A**) There was no significant difference between cats treated with CpG ODN 2216 vs. placebo at any of the four time-points. (**B**) Considering the entire population of cats, survivors had significantly higher *Mx* transcription at day 3 than non-survivors (*p* = 0.008). *Mx* transcripts significantly changed over time in survivors (*p* = 0.046; Dunn’s multiple comparison test not significant). Adm, admission; D1, day 1; D3, day 3; D7, day 7.

Considering the entire population of cats, *Mx* transcription significantly changed over time in survivors (*p* = 0.046; Dunn’s multiple comparison test not significant) and was significantly higher at day 3 in survivors compared to non-survivors (*p* = 0.008) (Figure 1).

On admission, all cats were viremic for FPV, and FPV DNA was identified in all fecal samples. The median viral loads in the blood and feces of the cats treated with CpG ODN 2216 and receiving placebo, on admission and during hospitalization, are reported in Table 5.

The median FPV loads in blood and fecal swabs gradually decreased throughout hospitalization in both groups. However, no significant differences were found within and between CpG ODN 2216 and placebo groups at any time-point.

**Figure 2:**
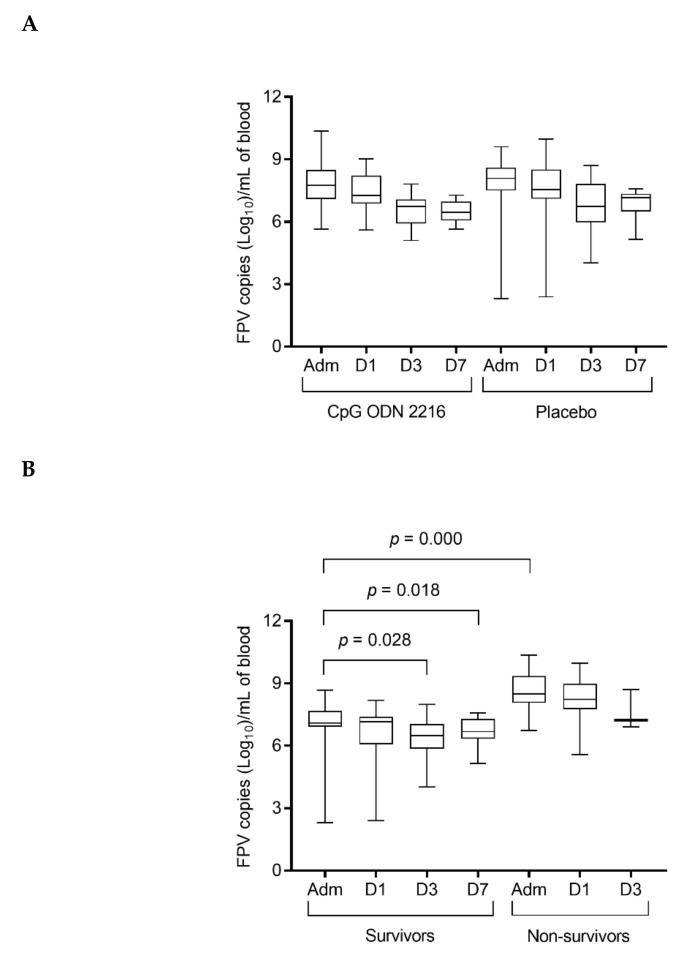
FPV (feline parvovirus) loads in peripheral blood (**A**) in cats treated with CpG ODN 2216 vs. placebo and (**B**) in survivors vs. non-survivors. Loads are given as log_10_ DNA parvovirus copies per mL of blood, as determined by real-time qPCR. Box and whisker plot: boxes give median and 25% and 75% quantiles; whiskers extend to min/max. (**A**) There was no significant difference between cats treated with CpG ODN 2216 vs. placebo at any of the four time-points. (**B**) Considering the entire population of cats, on admission, survivors had significantly lower FPV loads compared to non-survivors (*p* = 0.000). FPV loads decreased significantly over time in cats that survived (*p* = 0.004; Dunn’s multiple comparison test, admission vs. day 3 *p* = 0.028 and day 7 *p* = 0.018). Adm, admission; D1, day 1; D3, day 3; D7, day 7.

Considering the entire population of cats, FPV loads in the blood were significantly lower on admission in survivors compared to cats that died during the study (*p* = 0.000). FPV loads in the blood decreased significantly over time in cats that survived (*p* = 0.004; Dunn’s multiple comparison test, admission vs. day 3 *p* = 0.028, and admission vs. day 7 *p* = 0.018) (Figure 2).

**Figure 3:**
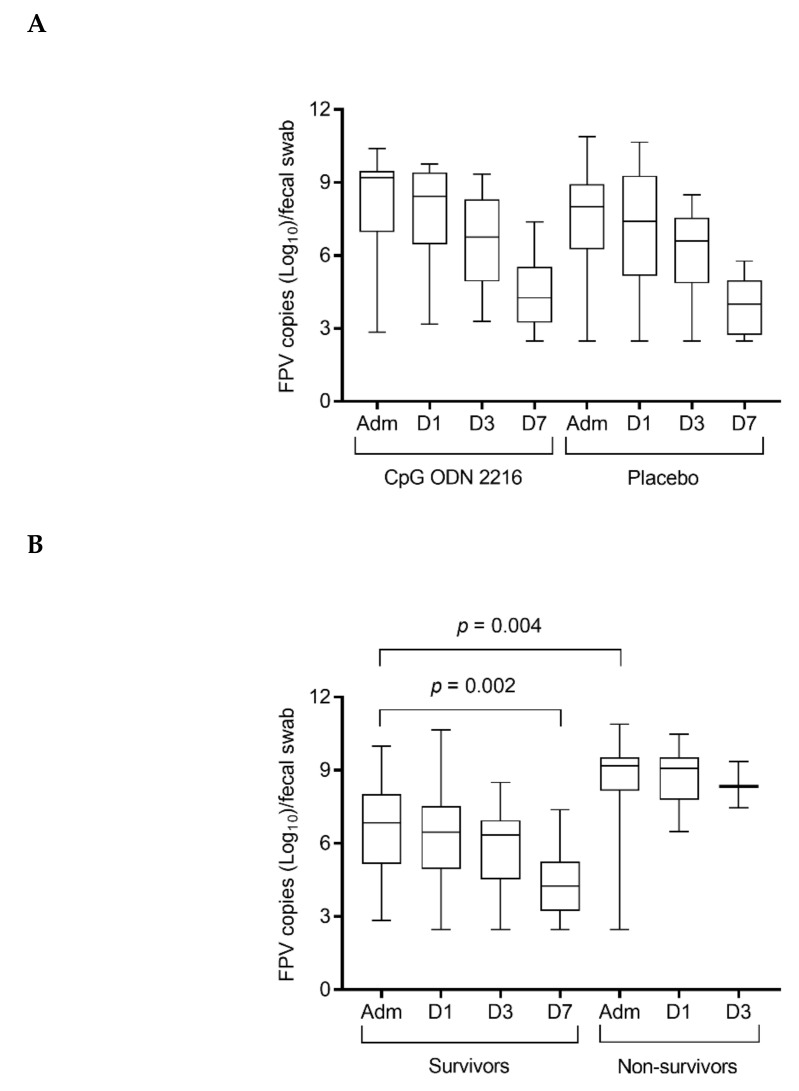
FPV loads in fecal swabs (**A**) in cats treated with CpG ODN 2216 vs. placebo and (**B**) in survivors vs. non-survivors. Loads are given as log_10_ DNA parvovirus copies per fecal swab, as determined by real-time qPCR. Box and whisker plot: boxes give median and 25% and 75% quantiles; whiskers extend to min/max. (**A**) There was no significant difference between cats treated with CpG ODN 2216 vs. placebo at any of the four time-points. (**B**) Considering the entire population of cats, on admission, survivors had significantly lower FPV loads compared to non-survivors (*p* = 0). FPV loads decreased significantly in cats that survived (*p* = 0.004; Dunn’s multiple comparison test, admission vs. day 7 *p* = 0.002). Adm, admission; D1, day 1; D3, day 3; D7, day 7.

Considering the entire population of cats, FPV loads in fecal swabs were significantly lower on admission in survivors compared to cats that died during the study (*p* = 0). FPV loads in fecal swabs decreased significantly in cats that survived (*p* = 0.002; Dunn’s multiple comparison test, admission vs. day 7 *p* = 0.002) (Figure 3).

Among the 42 cats of the study, 38 were tested on admission for FPV antibodies; in one cat of the CpG ODN 2216 group and three of the placebo, the collected blood sample was not enough for analysis. Thirteen (34.2%) of the tested cats had a positive immunofluorescence assay, while, in the remaining 25 (65.8%), it was negative. The median age of cats with a positive immunofluorescence assay was 4.0 months (interquartile range: 3.0–24.0); in particular, 2 cats were 2-month-old. Among those with FPV antibodies, nine (69.2%) belonged to the CpG ODN 2216 group and four (30.8%) to the placebo. The titer of the 13 positive cats was 1:20 in four (30.8%), 1:40 in three (23.1%), and 1:80 in six (46.1%). Among the 13 positive cats, 12 (92.3%) survived and 1 (7.7%) died; among the 25 negative cats, 7 (28.0%) survived and 18 (72.0%) died. A positive association was identified between the presence of antibodies against FPV on admission and survival (*p* = 0.002).

## 4. Discussion

In the present study, FPV infection induced a significant increase of *Mx* gene transcription on day 3 compared to admission in cats treated with either CpG ODN 2216 or placebo, but unexpectedly differences between the two treatment groups were not documented. Viral infections directly increase type 1 IFNs production by infected cells, causing a potent stimulation of both innate and adaptive immunity. IFNs induce the production of several molecules, which are able to prevent or inhibit viral replication and spread. Examples of these molecules are the 2′-5′ oligoadenylate synthetase, protein kinases, and Mx proteins [29]. In particular, the Mx proteins have been shown to have effective antiviral activity against bunyaviruses, orthomyxoviruses, paramyxoviruses, rhabdoviruses, togaviruses, picornaviruses, reoviruses, and hepatitis B virus in human and mice [29] and to reduce avian influenza H5N1 virus receptors in alveolar tissues of humans, macaques, ferrets, and cats [30]. Previous in vitro and in vivo studies in healthy cats have demonstrated the ability of CpG ODN 2216 to stimulate innate immunity against viral infections through activation of plasmacytoid dendritic and natural killer cells [10]. However, to the authors’ knowledge, the present was the first study that explored the effects of CpG ODN 2216 in cats naturally infected with FPV, using a treatment protocol based on healthy animals [10]. Hence, it could not be excluded that the dose and the frequency of administration of CpG ODN 2216 had to be increased in cats with FPV in order to stimulate an effective antiviral response and possibly determine significant improvements in survival, clinical signs, viremia, and viral shedding. It was probable that the severe leukopenia caused by FPV infection reduced the potential beneficial effects of CpG ODN 2216 since its antiviral activity was mediated by the activation of both humoral and cell-mediated immune response. Conversely, the immune-stimulatory effects of CpG ODN 2216 previously documented in healthy cats might be explained by the fact that their leukocyte counts were not decreased [10]. The scarce prevalence of FIV and FeLV infections did not allow to draw conclusions on the impact of these viruses on the immune response in the considered cat population. Whether FIV and FeLV infections might influence the effects of CpG ODN 2216 in FPV naturally infected cats needs further investigations.

Different clinical forms of feline panleukopenia are reported in the literature. The hyper-acute and acute forms are most commonly associated with high mortality rates, with fatalities occurring within 12 h in the former and 3–4 days in the latter [1]. Robert-Tissot and co-workers [10] demonstrated a significantly increased level of *Mx* transcription already 6 h after injection of CpG ODN 2216, which continued to rise for the first 2 days. Despite the rapid effect of CpG ODN 2216, it is also possible that the extremely fast progression of feline panleukopenia outweighed its potential beneficial effect in the present study, particularly in cats with hyperacute and acute forms. Moreover, a delayed institution of the treatment could have been responsible for a further reduction of its activity since, when cats show clinical signs (e.g., leukopenia and diarrhea), intestinal and immune system cells are already markedly damaged.

Clinical signs showed by the cats enrolled in this study were consistent with the ones reported in the literature in cats with parvovirus infection [1]. In the present study, the treatment with CpG ODN 2216 did not determine any significant improvement in clinical signs or in the clinical scores. Moreover, no significant changes in the viremia and viral shedding related to the treatment were observed.

According to the literature, complement proteins and antibodies can neutralize free virions and destroy virus-infected cells in human and animal viral infections. However, innate immunity and antibody-mediated immunity have a minor role in controlling viral diseases compared to cell-mediated responses. It is interesting to note that, in the present study, the presence of antibodies against FPV in serum samples collected on admission was positively associated with survival, with higher loads on admission in cats that died compared to survivors; similar results have already been described in the literature in cats infected with feline herpesvirus-1, feline calicivirus, and FPV [31]. Considering that one inclusion criterion was that cats were not vaccinated for FPV, the presence of antibodies on admission might suggest a previous contact with the virus or false positives due to nonspecific binding to the test reagents [31]; however, these hypotheses are less probable since the presence of antibodies is a strong predictor of protection against FPV reinfection and subsequent clinical disease, and since the slides for immunofluorescence assays were tested for potentially contaminating other feline viruses [2,3,4,5,6,7,8,9,10,11,12,13,14,15,16,17,18,19,20,21]. Because all cats came from a shelter where FPV is endemic, it is more likely that cats with neutralizing antibodies were admitted at a later stage of the infection; in fact, it has been shown that FPV antibodies production starts 8 days post-infection and precedes leukocytes improvement by several days [32]. In addition, in the two 2-month-old cats with positive FPV titer, an interference from maternal antibodies acquired via colostrum cannot be excluded [1].

Of note, in this study, cats with FPV that survived to discharge from the hospital had lower parvovirus loads in the peripheral blood and feces compared to cats that died and, by the end of hospitalization, experienced a further reduction of the viral load. These results were not unexpected and implied that cats with the overt disease but not overwhelmed by the virus had a favorable outcome. In addition, the data might suggest that qPCR could be used as a prognostic marker. However, in most clinical settings, achieving the results of qPCR usually requires several days, making the test less practical for prognostic purposes.

Vocalizations of a few seconds had been observed in all cats following CpG ODN 2216 injection; this reaction might indicate that cats experienced the pain of short duration in association with its administration, while it did not occur in the placebo group. Whether the administration procedures, such as the temperature of the CpG ODN 2216 solution, might have influenced the pain perception needs further investigations. No other adverse effects or local reactions to the injection were otherwise observed in any of the cats.

The main limitation of this study was the inability to precisely evaluate the amount of FPV-DNA in blood and feces due to the severe leukopenia and diarrhea, respectively. The DNA load was, therefore, normalized to the volume of blood and feces. Further investigations are needed to assess whether different doses of CpG ODN 2216 or classes of CpG, which have been shown to induce humoral response as vaccine adjuvant in mice, ducks, and primates [33,34], might be useful to treat cats during outbreaks of FPV.

## 5. Conclusions

In conclusion, CpG ODN 2216 administration in cats with naturally occurring FPV infection did not improve the clinical score, leukocyte and erythrocyte counts, did not decrease viremia and viral shedding, and did not improve outcome. FPV infection induced an antiviral response, but CpG ODN 2216 administration had no additional effect on the increase of *Mx* gene transcription in affected cats.

## Figures and Tables

**Table 1:** Four clinical parameters were combined to calculate the clinical score of cats with feline parvovirus (FPV) infection. The clinical score was given by the sum of the 4 assigned values. Values are reported in ascending order of severity.

Clinical Parameter	Value	Description
Attitude	0	Normal
	1	Depressed
	2	Lethargic
	3	Comatose
Appetite (% are in relation to the daily RER *)	0	75–100% RER
	1	50–75% RER
	2	25–50% RER
	3	Anorexia
Vomiting (number of episodes per day)	0	No vomiting
	1	Single episode
	2	Two episodes
	3	Three or more episodes
Feces	0	Normal feces or no feces
	1	Soft feces
	2	Diarrhea
	3	Watery diarrhea
	4	Hemorrhagic diarrhea

* RER: resting energy requirement.

**Table 2:** The median and interquartile range of clinical scores of the cats with FPV infection, treated with CpG ODN 2216 and receiving placebo, on admission and during hospitalization.

Time-Points	CpG ODN 2216	Placebo
Admission	6.0 (5.0–7.5)	7.5 (3.3–9.5)
Day 1	7.5 (6.0–9.7)	6.5 (2.0–11.0)
Day 2	7.0 (5.0–8.0)	5.0 (3.0–7.0)
Day 3	6.0 (3.2–10.0)	4.0 (1.5–5.0)
Day 4	4.0 (1.0–10.0)	2.5 (1.2–4.7)
Day 5	2.0 (0–5.5)	0.5 (0–3.5)
Day 6	0 (0–3.0)	1.0 (0–2.0)
Day 7	0 (0–1.0)	1.0 (0–2.7)

**Table 3:** The median and interquartile range of leukocyte and erythrocyte counts of the cats with FPV infection, treated with CpG ODN 2216 and receiving placebo, on admission and during hospitalization.

Time-Points	CpG ODN 2216		Placebo	
	Leukocytes (×10^3^/µL)	Erythrocytes (×10^9^/µL)	Leukocytes (×10^3^/µL)	Erythrocytes (×10^9^/µL)
Admission	1.6 (0.7–3.8)	6.5 (5.2–8.3)	0.8 (0.3–5.4)	6.9 (5.8–7.5)
Day 1	1.7 (1.0–4.1)	6.2 (5.2–8.5)	2.0 (0.3–4.1)	5.7 (5.1–6.5)
Day 3	6.3 (2.6–16.7)	5.7 (5.0–7.6)	8.6 (2.4–15.4)	5.5 (4.7–6.1)
Day 7	22.1 (18.0–33.3)	5.4 (4.3–7.4)	15.3 (9.1–20.1)	5.8 (5.3–6.4))

Leukopenia was defined as a leukocyte count <3500/µL, and anemia as a hematocrit <27%.

**Table 4:** The median and interquartile range of myxovirus (*Mx*) transcription of cats with FPV infection, treated with CpG ODN 2216 and receiving placebo, on admission and during hospitalization.

Time-Points	CpG ODN 2216	Placebo
Admission	0.5 (0.3–1.6)	0.4 (0.2–3.2)
Day 1	0.5 (0.2–4.0)	0.8 (0.4–4.6)
Day 3	3.0 (1.3–5.7) *	1.8 (1.0–5.6) *
Day 7	2.4 (1.1–7.3)	1.9 (0.7–3.7)

(*) *p* = 0.005, day 3 vs. admission and day 1.

**Table 5:** The median and interquartile range of FPV loads in the blood (expressed as log_10_ DNA parvovirus copies/mL of blood) and feces (expressed as log_10_ DNA parvovirus copies/fecal swab) of cats with FPV infection treated with CpG ODN 2216 and receiving placebo, on admission and during the hospitalization.

Time-Points	CpG ODN 2216		Placebo	
	Blood	Feces	Blood	Feces
Admission	7.7 (7.1–8.6)	9.2 (7.0–9.5)	8.1 (7.5–8.6)	8.0 (6.4–8.9)
Day 1	7.3 (6.9–8.2)	8.4 (6.5–9.4)	7.6 (7.1–8.5)	7.4 (5.3–9.3)
Day 3	6.7 (5.9–7.1)	6.7 (4.9–8.3)	6.7 (6.2–7.8)	6.6 (5.3–8.0)
Day 7	6.4 (6.1–7.0)	4.3 (3.2–5.5)	7.2 (6.5–7.3)	4.1 (2.9–5.0)
